# Utilizing Viral Metagenomics to Characterize Pathogenic and Commensal Viruses in Pediatric Patients with Febrile Neutropenia

**DOI:** 10.3390/v17030345

**Published:** 2025-02-28

**Authors:** Anielly Sarana da Silva, Gabriel Montenegro de Campos, Gabriela Marengone Altizani, Enéas de Carvalho, Alice Chagas Barros, Eleonora Cella, Simone Kashima, Sandra Coccuzzo Sampaio, Maria Carolina Elias, Marta Giovanetti, Carlos Alberto Scrideli, Svetoslav Nanev Slavov

**Affiliations:** 1 Blood Center of Ribeirão Preto, Faculty of Medicine of Ribeirão Preto, University of São Paulo, Ribeirão Preto 14051-140, Brazil; anielly.sarana@gmail.com (A.S.d.S.); gabrielmdecampos@usp.br (G.M.d.C.); skashima@hemocentro.fmrp.usp.br (S.K.); 2 Department of Puericulture and Pediatrics, Faculty of Medicine of Ribeirão Preto, University of São Paulo, Ribeirão Preto 14049-900, Brazil; gmaltizani@hcrp.usp.br (G.M.A.); scrideli@fmrp.usp.br (C.A.S.); 3 Butantan Institute, Avenida Vital Brasil, 1500, São Paulo 05503-001, Brazil; eneas.carvalho@butantan.gov.br (E.d.C.); sandra.coccuzzo@butantan.gov.br (S.C.S.); carolina.eliassabbaga@butantan.gov.br (M.C.E.); 4 Central Laboratory, University Hospital of the Faculty of Medicine in Ribeirão Preto, University of São Paulo, Ribeirão Preto 14040-030, Brazil; acbarros@hcrp.usp.br; 5 Burnett School of Biomedical Sciences, College of Medicine, University of Central Florida, Orlando, FL 32827, USA; eleonora.cella@yahoo.it; 6 Sciences and Technologies for Sustainable Development and One Health, Università Campus Bio-Medico di Roma, 00128 Rome, Italy; giovanetti.marta@gmail.com; 7 Instituto Rene Rachou, Fundação Oswaldo Cruz, Belo Horizonte 30190-002, Brazil; 8 Center for Viral Surveillance and Serological Evaluation (CeVIVas), Butantan Institute, Avenida Vital Brasil 1500, São Paulo 05503-900, Brazil

**Keywords:** metagenomics, virome, herpesviruses, febrile neutropenia, oncology, pediatrics

## Abstract

Febrile neutropenia (FN) is one of the most common complications in pediatric oncology patients. It has a complex etiologic nature, which in the majority of cases remains unclear. Intervention often follows empirical treatment protocols, mainly using broad-spectrum antibiotics. To evaluate potential viral etiologic agents, this study applied viral metagenomics to paired plasma and oropharyngeal samples obtained from pediatric patients with oncological diseases diagnosed with FN. Metagenomic sequencing was performed on 15 pediatric patients with oncological diseases and FN at the outpatient clinic of Pediatric Oncology at the University Hospital of the Faculty of Medicine of Ribeirão Preto, University of São Paulo. As a control group, we included 15 pediatric patients with oncological diseases in remission or undergoing treatment. Clinically relevant viruses identified by metagenomics in FN patients predominantly included herpesviruses and viruses found in the respiratory tract, like adenoviruses. Direct molecular confirmation was performed on all of them. Anelloviruses, represented by various genera and species in all groups, were also highly prevalent. The data obtained in this study show that viruses might also have possible implications for the etiology of FN. However, due to the complex nature of this disease, more studies are necessary to evaluate their causal relationship. The results obtained in our study may serve to improve patient treatment and ensure adequate management.

## 1. Introduction

Febrile neutropenia (FN) is a common and serious complication of myeloablative chemotherapy that is characterized by fever and a sudden decrease in the absolute neutrophil count (ANC) to below 500 cells/mm^3^ [1,2,3,4]. FN is most often associated with conditions such as acute myeloid leukemia (AML), acute lymphoblastic leukemia (ALL), lymphomas, myelomas, and certain solid tumors requiring intensive care [3,5,6]. The treatment protocol for FN involves the administration of broad-spectrum antibiotics, with a primary focus on cephalosporins (such as cefepime) and carbapenems (such as imipenem/cilastatin) [7].

The most commonly implicated etiological agents for FN are Gram-positive bacteria, including *Staphylococcus*, *Streptococcus*, and *Enterococcus* species, as well as multi-drug-resistant bacteria such as *E. coli*, *Pseudomonas aeruginosa*, and *Klebsiella* spp. [3,4]. Although rare, viral agents have also been associated with cases of FN, including members of the *Herpesviridae* family such as Epstein–Barr virus (EBV) [8] and herpes simplex virus-1 (HSV-1) [9], as well as a variety of respiratory viruses including influenza, parainfluenza [10], rhinoviruses [11], human adenoviruses (hADV) [12], severe acute respiratory syndrome coronavirus 2 (SARS-CoV-2) [13], and respiratory syncytial virus (RSV) [14]. However, the etiology of FN in most cases remains unknown, as bacterial cultures are positive in fewer than half of cases [15,16].

Given the diverse range of pathogenic agents that can contribute to FN and considering also the limitations of targeted detection methods such as bacterial culture, we employed metagenomic next-generation sequencing (mNGS) to evaluate the virome in pediatric patients presenting with FN who lacked an etiological diagnosis via standard bacterial culture. mNGS, along with metagenomic analysis, provides an unbiased approach to sequencing, offering a comprehensive view of all genetic material present in a clinical sample. This method has been widely used in clinical practice, particularly for the detection of unknown or unsuspected pathogens [17]. Characterizing the virome in these patients and specifically identifying potential pathogens is crucial not only for understanding the disease’s etiology but also for developing strategies for effective treatment and management.

Thus, the objective of this study was to analyze the total virome of 15 pediatric patients with oncological diseases presenting with FN at the outpatient clinic for pediatric oncological diseases at the Department of Pediatrics and Puericulture, University Hospital of the Faculty of Medicine of Ribeirão Preto, University of São Paulo. The results obtained were compared with a control group composed of 15 pediatric patients with oncological diseases undergoing treatment, in remission, or attending routine consultations.

## 2. Materials and Methods

### 2.1. Study Design

Paired blood and oropharyngeal samples (samples collected from the oropharynx, primarily from the cheek mucosa) were collected from 15 children presenting with FN at their admission to the Pediatric Oncology Clinic at the University Hospital of the Faculty of Medicine of Ribeirão Preto (HC-FMRP), between April and September 2023. Concurrently, a control group was established, comprising 15 pediatric patients with oncological diseases either undergoing treatment or in remission. The patients belonging to the control group periodically returned to the same clinical unit for routine follow-up examinations. Our study was designed as a case–control investigation, enabling comparison between groups with and without the condition of interest. This type of design is suitable for examining specific associations at a given point in time.

The characteristics of the included patients are shown in Table 1.

Participation in the study was contingent upon obtaining written informed consent from the patients’ legal guardians. The study received approval from the Institutional Ethics Committee Board of the University Hospital of the Faculty of Medicine of Ribeirão Preto, University of São Paulo, under the number 66508422.0.0000.5440.

### 2.2. Sample Processing and Illumina Sequencing

Total nucleic acids from individual plasma and oropharyngeal swab samples were extracted using a High Pure Viral Nucleic Acid Large Volume Kit (Roche, Basel, Switzerland), following the manufacturer’s instructions with several modifications, as follows: (1) For removal of host and bacterial DNA, individual samples were initially pre-treated with 20 U of turbo DNase (ThermoFisher Scientific, Waltham, MA, USA); (2) to ensure the concentration of nucleic acids, we used GenElute Linear Polyacrylamide carrier (LPA) (Merck, Darmstadt, Germany); and (3) precipitation was performed with −20 °C absolute isopropanol. Reverse transcription was performed using a Superscript III First-Strand Synthesis System (ThermoFisher Scientific, Waltham, MA, USA). cDNA and DNA were amplified isothermally using a QuantiTect Whole Transcriptome Kit (QIAGEN, Hilden, Germany) with the following protocol. (1) Single-strand ligation reaction was performed for 2 h at 22 °C; and (2) ligand amplification was performed during isothermal amplification for 8 h at 30 °C, using REPLI-g Midi Reaction Buffer and REPLI-g Midi DNA polymerase.

Metagenomics libraries were prepared using a DNA Prep Library Preparation Kit (Illumina, San Diego, CA, USA), adhering to the manufacturer’s instructions, with tagmentation input of 500 ng. Indexing was carried out using IDT for Illumina DNA/RNA UD Indexes. Pair-end sequencing of the dual-indexed libraries was performed on the Illumina NextSeq 2000 sequencing platform using a NextSeq P3 flow cell system (300 cycles) (Illumina), following the manufacturer’s instructions.

### 2.3. Bioinformatic Processing of the Raw Sequencing Data and Taxonomic Classification of Viral Reads

The obtained raw sequence data were submitted to quality control analysis using FastQC v.0.11.8 software [18]. Trimming and adapter removal were performed applying Fastp v.0.23.1 [19] to select sequences with the best quality that were free of adapters. For the metagenomic analysis, we used only reads with a quality score >30. Burrows–Wheeler Aligner v.1.0.3 software was used to filter and subtract human genome reads [20]. To infer the taxonomic classification of the virome, we used Kraken2 v.2.0.8 [21] with application of the NCBI Virus databank. Non-viral reads, reads from viruses that do not cause human infections, phages, and artifacts were manually removed from the Kraken2 output, as they were not objects of interest in this study. Sampling after manual curation of the virome included viruses of clinical interest and commensal viruses (different anelloviruses and HPgV-1), which were used for further analysis.

### 2.4. Analysis of the Viral Diversity

The viral abundance was estimated based on the total number of raw read counts obtained in the Kraken2 results after filtering, along with the normalization of these reads with log2 transformation. In addition to this, the Kraken2 results were transformed into tables to better visualize the reads by patient and by viruses found. The tables were plotted in bar graphs using the RStudio environment (v.4.2.3), in R programming language, using the ggplot2 v.3.5.1 package [22] and heatmap3 v.1.1.9 [23]. We used the packages phyloseq v.3.20 and vegan v.2.5.7 to calculate and plot the Shannon and Simpson indices of diversity and the rarefaction curves. Principal component analysis (PCA) was performed using the package factoextra [24,25].

The abundance of anelloviruses was estimated using Bracken v.2.9 (Bayesian Reestimation of Abundance with Kraken) [26], which can produce very accurate estimates even when analyzing many similar viral species, especially regarding anelloviruses. The Shannon diversity index (H’) among anelloviruses in the tested groups was calculated using the microeco package v. 1.13.0 [27].

### 2.5. Direct Detection of Clinically Important Viruses in Individual Samples

We confirmed the following individual viruses, principally including herpesviruses: EBV, human cytomegalovirus (CMV), human herpesvirus 6 and 7 (HHV-6 and 7), and HSV-1. We also confirmed hADV and the polyomaviruses Malawi (MWPyV) and Washington University (Saint Louis) virus (WUPyV). Conventional nested PCR was applied only to hADVs, following a well-established protocol targeting amplification of a conserved region within the hexon gene [28]. Other viral agents were detected using real-time PCR with primers and probes as described in the literature. The sequences and the respective concentrations of the used primers and probes are shown in Table 2.

Except for hADV, the real-time molecular confirmation of the rest of the viruses included qPCR using a qPCR GoTaqR One-Step RT system (Promega, Madison, WI, USA), without the addition of reverse transcriptase, as all of the tested viruses presented a DNA genome. The reaction involved 1X de GoTaq^®^ qPCR Master Mix, 0.33 µL of CXR Reference Dye, the respective concentrations of the primers and the probes (see Table 1), and at least 100 ng of DNA, within a final volume of 20 µL. The thermal cycling was conducted under the following protocol: an initial step at 50 °C for 2 min, followed by denaturation at 95 °C for 10 min, and 40 cycles consisting of 95 °C for 15 s and 60 °C for 1 min. All the reactions were performed in an Applied Biosystems 7500 cycler (ThermoFisher Scientific, Waltham, MA, USA) with automatic extrapolation of the cycle threshold (Ct) for the amplification of each virus.

The hADV detection reaction used conventional nested PCR and was performed in a final volume of 50 μL, consisting of 10X PCR Buffer, 3 mM of MgCl_2_, 0.25 mM of dNTPs, 2 U of Platinum Taq DNA Polymerase (ThermoFisher Scientific) and ~100 ng of extracted viral nucleic acid. Cycling conditions included initial denaturation at 94 °C for 3 min, followed by 40 cycles of 95 °C for 30 s, 55 °C for 30 s, and 72 °C for 1 min, and a final extension for 5 min at 72 °C. Amplification was performed in a SimpliAmp Thermal Cycler (ThermoFisher Scientific, Waltham, MA, USA). For the second reaction, 4 μL of the amplification product obtained in the first reaction was applied at the same concentration of reagents and cycling conditions as the first reaction, apart from the cycle number (35 cycles). Amplicon visualization was performed in 2% agarose gel stained with GelRed^®^ Nucleic Acid Gel Stain (Biotium, Hayward, CA, USA) and visualization was performed on ChemiDoc XRS+ using Image Lab Software v.3.0.1 (Bio-Rad, Hercules, CA, USA). All pre- and post-amplification steps were carried out in separate laboratory rooms to avoid contamination.

### 2.6. hADV Confirmation by Ampicon Sequencing

hADV-positive samples were submitted for confirmation by Sanger sequencing of the material obtained from the second nested-PCR amplicon of 171 bp. Sequencing was performed in an ABI 3500 XL DNA sequencer (ThermoFisher Scientific, Waltham, MA, USA) with a BigDye™ Terminator v.3.1 Cycle Sequencing Kit (ThermoFisher Scientific, Waltham, MA, USA), using 1 μM of forward primer from the second nested PCR. The cycling included initial denaturation at 95 °C for 1 min, 25 cycles of 96 °C for 10 s, 50 °C for 5 s, and 60 °C for 4 min. The obtained sequence was aligned in BLASTn (NCBI) for confirmation of the hADV type [36].

### 2.7. Statistical Analysis

Logistic regression was performed on the viral diversity obtained from oropharyngeal swabs, with FN and herpesvirus reads as dependent and independent variables, respectively. The binary data transformation considered FN as 1 and the control group as 0, and patients with any number of reads from herpesviruses as 1 and those with no reads as 0. Results were considered significant when *p*-value < 0.05. The statistical analyses were performed using R v.4.2.2 [37]. The post hoc power analysis indicated a statistical power of 80% to allow the detection of a difference of at least 0.4 between the two groups. The analysis was conducted using Stata v. 10.4.

## 3. Results

### 3.1. Sequencing Results: Quantitative Results

mNGS was carried out on individual samples to obtain more detailed and high-depth information regarding the virome abundance for each unique patient.

The sequencing generated 355.54 Gbp of data, with a Q30 score in 84.18% of the obtained reads. A total number of 1,995,917,632 raw sequence reads were obtained, which, after trimming and adapter removal, totaled 1,420,954,929. After subtracting human-origin sequences, the remaining 300,933,078 reads were classified by Kraken2 (21.18%). Among the classified sequences, 11.98% were of viral origin (36,052,338 reads). Unclassified sequences represented 7% of the total reads after trimming (99,668,071). Detailed information on the quantitative sequencing data by group can be viewed in Table 3.

FN patients showed a higher percentage of viral reads in plasma, in comparison with the paired swab samples. In the control group, which included children with cancer in remission, the percentages of viral reads in plasma and swab were similar.

### 3.2. Viral Diversity

**Figure 1:**
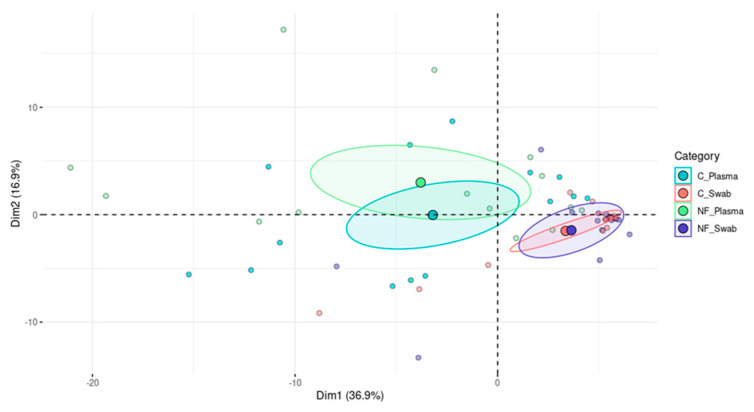
Filtered virome, principal component analysis. NF: Febrile neutropenic patients’ virome; C: Control group patients’ virome. The virome appears to cluster based on the sample origin (swab or plasma), regardless of febrile neutropenia presence.

Figure 1 shows the results of the principal component analysis (PCA) applied to the patient data in this study, based on the normalized reads of the filtered virome. The analysis revealed that clustering was predominantly influenced by the sample origin (plasma or swab) rather than by the diagnosis of FN. As indicated in Figure 1, Dim2 showed higher variability in the virome among plasma samples linked to the diagnosis of FN than in the oropharyngeal swabs. In the swab samples, despite similar dispersion between groups, the FN group showed higher diversity in Dim2 than the control. Dim1 appeared similarly distributed between samples, and the plasma samples presented greater diversity between the groups. In summary, the virome of the patients in this study tended to group together based on sample origin, and the plasma virome seems to have been more diversified than that of the swab samples, considering the FN and control groups.

**Figure 2:**
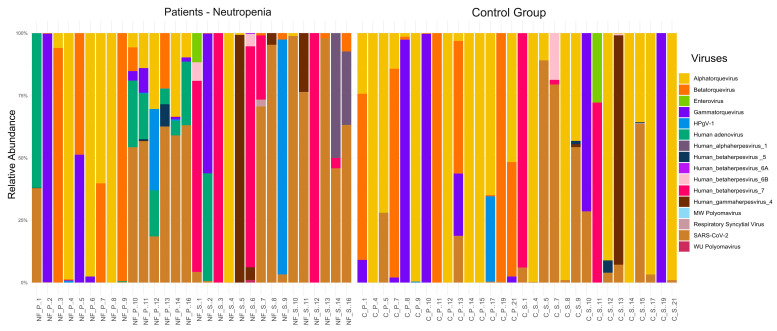
Relative abundance of viruses at species and genus level found in patients in the control group and in the febrile neutropenic group, % of reads. Each individual sample refers to plasma (P) or saliva (S). Multiple herpesviruses that were identified in high read numbers in saliva of patients with febrile neutropenia were consequently confirmed by qPCR. In comparison, their abundance in patients without febrile neutropenia was much lower.

Assessment of relative viral abundance resulted in the identification of representative viruses, as can be observed in Figure 2. Anelloviruses of the alpha, beta, and gamma torque virus genera were highly abundant, indicating the presence of torque teno viruses (TTV), torque teno mini viruses (TTMV) and torque teno midi viruses (TTMDV), respectively. The high number of reads corresponding to beta coronaviruses and more specifically, SARS-CoV-2 belonged to a single patient, FN6, who tested positive for SARS-CoV-2 in the hospital during the collection period. Viruses belonging to the *Anelloviridae* family were predominant across all tested groups and clinical samples, except for the swab samples obtained from febrile patients with neutropenia, where EBV was the majority.

In plasma samples, among the viruses with clinical importance identified in patients with FN, the most prevalent were representatives of the *Herpesviridae* family (HSV-1, CMV, HHV-6, HHV-7, and EBV) and hADV (patients FN8 and FN10). A high number of CMV reads were observed in Patient FN7.

In plasma samples from the control group, approximately 99.4% of the total number of reads of viruses corresponded to anelloviruses. TTV representatives and HPgV-1 were particularly abundant.

The viral abundance observed in swab samples differed considerably from that in plasma. In the group of pediatric patients with FN, anelloviruses represented only 10.7% of the viral abundance. In this group, similar to the observations for plasma, viruses from the *Herpesviridae* family were the most predominant, with more than 80% of the reads classified as belonging to EBV (patients FN5, FN14, and FN16). Other herpesviruses that were also identified included HSV-1, HHV-6, and HHV-7, at lower read counts. hADV types 1 and 5 were found in patients FN8 and FN11, respectively. Interestingly, low numbers of sequence reads corresponding to two lesser known polyomaviruses were inferred by Kraken: MWPyV in patient FN15 and WUPyV in patient FN16. Coxsackievirus A16 (also known as hand-foot-and-mouth disease virus) was identified in patient FN14. In swab samples obtained from the control group patients, 85% of all classified viral reads indicated representatives of the *Anelloviridae* family. In this group, we also found sequence reads from material belonging to the *Herpesviridae* family, especially CMV in patient FNC10, HHV-6 in patient FNC13, and a high number of HHV-7 reads in patient FNC10, all of which were consequently confirmed by direct molecular methods.

Considering the different viral landscape observed in the swab samples, we decided to analyze the relationship between the presence of reads of herpesviruses (>0) and the probability of these patients being diagnosed with FN. We conducted logistic regression of these data after transforming them into binary variables, and the results indicated that FN did not have any significant effect regarding the presence of herpesviruses reads in the swabs of patients in this study (OR = 1.0, *p*-value > 0.05).

**Figure 3:**
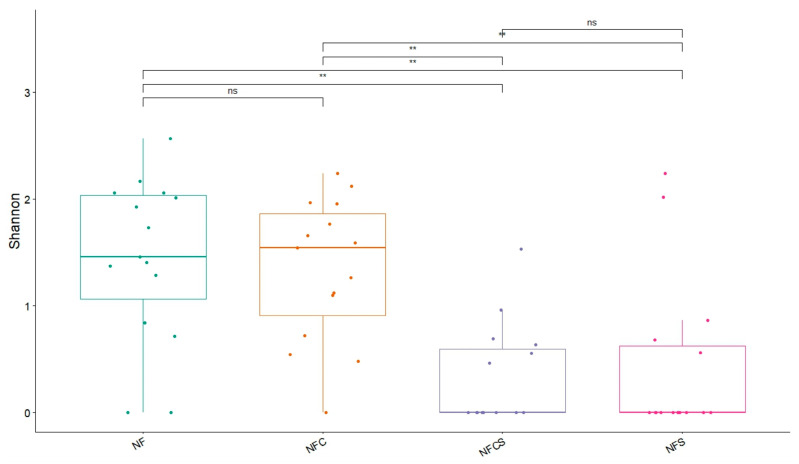
Shannon diversity index for anelloviruses present among both groups. NF: febrile neutropenic patients, plasma samples; NFC: control group, plasma samples; NFCS: control group in swab samples; NFS: febrile neutropenic patients, swab samples. ns: not significant. Results were considered significant when *p*-value < 0.05. **: *p*-value < 0.01.

Observing the expressive anellovirus abundance across all groups, we decided to analyze whether their conjunction diverged with regard to diagnosis of FN. For this purpose, we applied Shannon diversity analysis, as shown in Figure 3. The Shannon index of the anelloviruses from samples of the same origin, regardless of the group, were very similar. For both groups, the plasma samples presented values between 1 and 2, while the swab samples’ diversity index scores were close to 0 for both groups. Anellovirus diversity was significantly higher in the blood (which was expected) compared with the respiratory tract (*p*-value < 0.01), considering individuals from the same group.

### 3.3. Direct Confirmation and Molecular Characteristics of the Identified Viruses via mNGS

#### 3.3.1. qPCR Detection of Herpesviruses

Direct detection of all viruses with presumed clinical significance was performed. Herpesviruses HSV-1, EBV, CMV, HHV-6, and HHV-7 were detected using real-time PCR and hADV via genus-specific nested PCR. Clinically important viruses were directly confirmed in the clinical samples by the presence of sequence reads obtained by mNGS irrespective of their number. Due to the increased interest in polyomaviruses and the presence of several reads belonging to this viral family, these were also confirmed. The viruses detected among patients with FN, including the type of clinical sample, Ct, and the number of reads detected via metagenomics is shown in Table 4.

**Figure 4:**
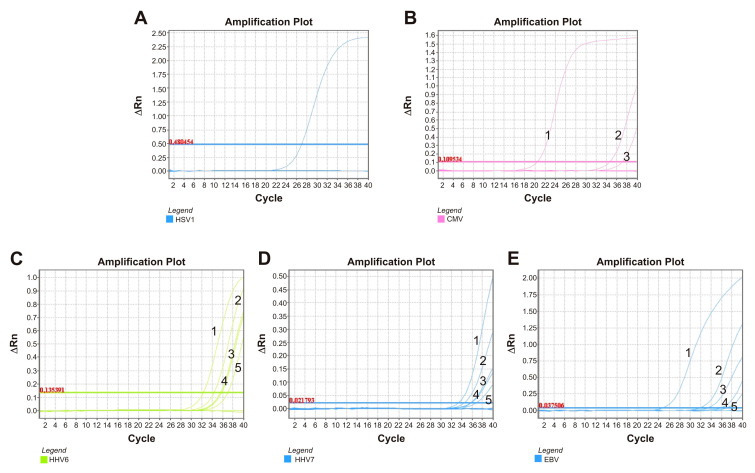
Amplification plots for the tested herpesviruses obtained by qPCR together with their automatic cycle thresholds in individual clinical samples obtained from pediatric patients with febrile neutropenia, as well as from the control group. (**A**): HSV-1, only the positive control is displayed, with Ct = 26.96. (**B**): CMV, numbers 1, 2, and 3 correspond, respectively, to the positive control (Ct = 20.51) and to the plasma of patients FN7 (Ct = 34.64) and FN5 (Ct = 36.99). (**C**): HHV-6, numbers 1, 2, 3, 4 and 5 correspond, respectively, to the positive control (Ct = 32.3) and the swabs from patients FN13 (Ct = 34.38), FN16 (Ct = 35.57), FNC10 (Ct = 35.81), and FN10 (Ct = 36.95). (**D**): HHV-7, numbers 1, 2, 3, 4, and 5 correspond, respectively, to the swabs from patients FNC10 (Ct = 33.61), FN16 (Ct = 34.68), FN1 (Ct = 35.9), FN12 (Ct = 36.02), and FN10 (Ct = 36.96). (**E**): EBV, numbers 1, 2, 3, 4, and 5 correspond, respectively, to the swabs from patients FN14 (Ct = 24.75), FN5 (Ct = 31.81), FN2 (Ct = 33.4), and FN16 (Ct = 35.86) and the plasma of patient FN5 (Ct = 37.15).

HSV-1 sequence reads were identified in eight patients, six with FN and two from the control group. In none of the clinical samples were we able to confirm HSV-1 DNA by qPCR. Amplification plots of HSV-1 can be observed in Figure 4A.

Sequence reads belonging to CMV were identified by metagenomics analysis in six patients and were directly confirmed in two of these in the plasma of patients FN5 and FN7. The Ct were high: 36.99 and 34.64, respectively. The confirmation of CMV by qPCR is shown in Figure 4B.

HHV-6 and HHV-7 were also tested by qPCR, and the amplification plots are shown in Figure 4C and Figure 4D, respectively. HHV-6 reads were present in swab samples of five patients, and qPCR showed positive results in four of them: FN10 (Ct = 36.95), FN13 (Ct = 34.38), and FN16 (Ct = 35.57) from the FN group and one patient from the control group, FNC10 (Ct = 35.81). All the samples showed similar Ct of HHV-6 with a mean sample Ct = 35.68. Regarding the presence of HHV-7 DNA, nucleic acids were detected by real-time PCR in 5 of 10 patients whose reads were identified by metagenomics. In all cases, HHV-7 was detected in swab samples. HHV-7 was confirmed in the control group in only one patient: FNC10, with a Ct of 33.61. Among the FN patients, it was confirmed in FN1 (Ct = 35.9), FN10 (Ct = 36.96), FN12 (Ct = 36.02), and FN16 (Ct = 34.6), mean Ct = 35.87.

The last herpesvirus confirmed by qPCR was EBV, which was identified via mNGS in seven pediatric patients. It was directly confirmed in five of them; in the FN group, it was confirmed in patient FN5, in both swab and plasma, with respective Cts of 31.81 and 37.15, and in the swabs of patients FN2 (Ct = 33.4), FN14 (Ct = 24.75), and FN16 (Ct = 35.86). Due to the relatively low Ct detected in the swab sample of patient FN14, accompanied by a substantial number of reads (more than one million) identified by mNGs, we believe that such infection might be regarded as acute. The amplification of the EBV plot can be observed in Figure 4E.

#### 3.3.2. qPCR Detection of Polyomaviruses MW and WU

**Figure 5:**
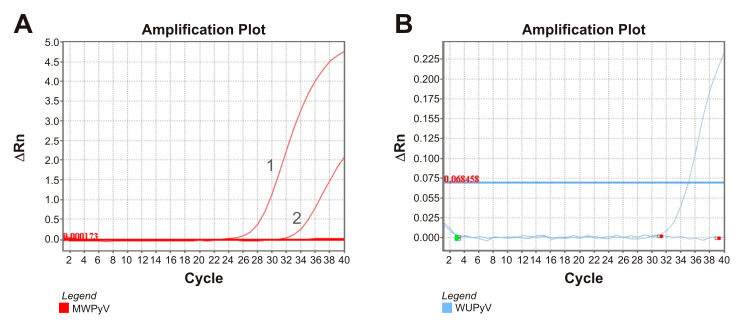
Nucleic acid amplification curves according to qPCR with automatic cycle threshold estimation in individual sample confirmation of the polyomaviruses MWPyV and WUPyV. (**A**): Amplification plot of MWPyV. Number 1 corresponds to the amplification curve of the MWPyV positive control, and number 2 corresponds to the swab of patient FN16, Ct = 25.98. (**B**): Amplification plot of WUPyV of the only sample that was identified by mNGS. The positive patient FN16 showed Ct = 34.

The polyomaviruses identified through metagenomic analysis were MW and WU (MWPyV and WUPyV), respectively, in the swab samples from patients FN5 (3 reads) and FN16 (17 reads). Despite the low numbers of reads, the amplification of MWPyV in individual samples unexpectedly rendered a Ct of 25.98. We suspected that mNGS performed on individual samples may reduce the individual coverage. Therefore, careful interpretation of the metagenomics data including observation of important viruses present in low viral reads is mandatory. The MWPyV amplification plot is shown in Figure 5A. The amplification of WUPyV was also positive. WUPyV is also a rare and emerging polyomavirus and its amplification plot is shown in Figure 5B, with an estimated Ct of 34.

#### 3.3.3. Nested PCR for Confirmation of hADV

**Figure 6:**
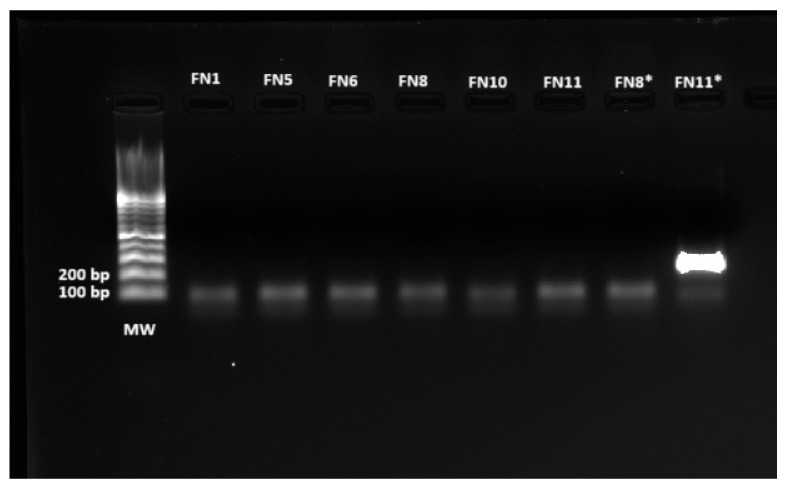
Electrophoresis in 2% agarose gel of the samples tested for hADV by nested PCR. The only positive amplification corresponded to a band obtained from patient FN11, with an approximate size of 171 bp. Consequently, the amplicon was sequenced and it was confirmed that it belonged to hADV type 30. MW: molecular weight marker; *: swab samples from the respective patients with febrile neutropenia.

The metagenomics analysis identified hADV reads in several samples, both swab and plasma, and these were classified at the level of the *Mastadenovirus* genus and at species level as hADV 1 and 5. Due to the high diversity of the hADV, we performed a well-established nested-PCR technique able to detect up to 51 hADV serotypes by amplification of a coding region of the hexon protein [28]. Amplicons were revealed in a 2% agarose gel, and the 171 bp amplicon was visualized only in the band belonging to the swab sample from patient FN11, as shown in Figure 6.

The positive swab sample from patient FN11 was submitted to Sanger Sequencing for determination of the hADV type. After performing BLAST alignment, it was revealed that the conserved region from the hexon gene presented 98.4% identity with hADV 30 strain Hu (E-value = 3 × 10^−25^; query cover = 91%; accession number MK296458.1).

## 4. Discussion

FN is regarded as a common adverse effect resulting from myelosuppressive chemotherapy, with a complex pathogenesis [3]. It can result from the interplay between the immune suppression on the background of opportunistic or pathogenic microbial agents, with emergency treatment based mainly on symptomatology [5]. By applying viral metagenomics in cases of pediatric patients with negative bacterial cultures, we were able to identify and confirm viral agents which could also have been implicated as possible causes for FN. In general, this helps us to understand the behavior of the viral component in this severe clinical condition, which is related to high lethality rates.

The plasma virome of the screened patients was mainly composed of commensal viruses, while in the swab, we observed a predominance of different herpesviruses. In previous studies that have described the FN microbiome, although they have not paid special attention to viruses, patients have usually presented a wide array of herpesviruses, including CMV, EBV, HHV-6, and HHV-7, and also respiratory-acquired viruses like hADV, influenza, parainfluenza, and RSV, among others [8,38]. Respiratory viruses pose an important threat to patients with immune suppression, due to rapid progression of infection and high mortality [3,11,39]. The literature data is compatible with our results; however, previous studies mainly focused on investigating plasma samples. In that respect, one difference of our investigation is that we collected paired samples from each patient with FN, i.e., oropharyngeal swabs and plasma. Additionally, mNGS was performed individually and not in sample pools, giving a better picture of the viral abundance in general.

In the oropharyngeal samples, mNGS identified a low number of reads belonging to two polyomaviruses, which were consequently confirmed by qPCR, that have never previously been reported in patients with this clinical condition. We believe that these polyomaviruses represent primary infections as they were acquired in early childhood [40]. However, due to the immune conditions of these patients, we do not have information on the dynamics of polyomavirus detection, as serial samples were not obtained from the tested groups. Little is known about the impact of these viruses on patients with FN. The identified MWPyV and WUPyV are emerging viruses with unknown clinical impact, including on patients with FN and immune suppression [33,41,42]. For that reason, we believe that more detailed investigations are needed in order to clarify whether these two identified polyomaviruses present causal relationships with states of neutropenia or are just bystanders revealed by deep-sequencing approaches.

The highest sequence read abundance was observed for the herpesviruses that were present in considerable numbers in FN patients and especially in nasopharyngeal swabs. Herpesviruses, especially CMV and EBV, are frequently reactivated among immunosuppressed patients and may cause systemic manifestations like pneumonia, gastrointestinal manifestations, retinitis, or hepatitis, among others, worsening clinical outcomes [43,44,45,46]. Although the relative viral abundance detected in FN swab samples may suggest that herpesviruses’ presence is more prominent in patients presenting this condition, the qualitative results provided by the logistic regression suggest that there is insufficient evidence to conclude that FN status impacts the clinical outcome. This is reasonable to suppose, as the two study groups presented different levels of immune suppression. In addition, the presence of herpesviruses revealed by metagenomics analysis, including their consequent confirmation by molecular methods, must be regarded with caution due to asymptomatic excretion of these viruses, including in saliva [47]. For that reason, complementary tests studying the dynamics of these infections by applying quantification of viral load against a background of clinical symptomatology are essential to confirm the clinical involvement of these viruses [48]. Therefore, in this study, we also assessed the molecular parameters of the infections expressed in the evaluation of the cycle threshold of amplification. In one case (swab from patient FN14), we observed EBV amplification with low Ct (24.75), corroborating the high number of EBV sequence reads obtained by metagenomics and possibly indicating active infection. Active EBV infection can lead to a condition known as infectious mononucleosis, related to a sudden drop in neutrophil count, with sore throat, fever, palatine petechiae, and lymphadenopathies as general symptoms [49,50]. Implicating EBV as a cause of FN is a complex matter that involves careful examination of clinical data and following the dynamics of the infection. As mentioned above, we collected unique samples from each patient and as a result, we were unable to follow the dynamics of EBV infection on the background of changes of clinical parameters. Nonetheless, pediatric patients with immune suppression experience very high rates of reactivation of herpesviruses and this, coupled with their potential to cause severe disease including neuroinvasive outcomes, poses critical issues among patients with FN [51,52]. Although viral encephalitis is not common in patients with FN, it has been previously reported and should be carefully considered both because it is potentially fatal and difficult to diagnose and because of the high prevalence of herpesviruses in the pediatric population [53].

In addition to herpesviruses, other viruses that were directly confirmed included some that commonly infect the upper respiratory tract: ADV and two types of polyomaviruses, MWPyV and WUPyV, discussed above. The COVID-19 pandemic has made it clear that the potential of respiratory viruses should not be underestimated, and this is particularly relevant due to the involvement of respiratory viruses in the etiology of FN [54,55,56]. Considering that the primary symptoms of FN in children usually manifest in the upper respiratory tract, it is not unexpected that viruses that cause respiratory infections were found in the patients in this study [10]. In the case of ADVs, Schulz and colleagues reported a case of acute hADV infection in an elderly patient with FN with a lethal outcome [57]. In studies with larger patient groups, the positivity rate for hADVs in pediatric patients during cancer treatment has reached 0.3% [58]. In the case observed in our study, the infection was initially identified by mNGS as soon as the patient presented FN. Consequently, hADV was confirmed in patient FN11’s oropharyngeal swab sample, and local alignment showed high similarity with the hADV 30 strain Hu from the subgroup D. This type of adenovirus is involved in keratoconjunctivitis and gastrointestinal infections [59].

An important observation in our study is that not all viruses detected by mNGS were confirmed by qPCR. Additionally, viruses with a low read count—particularly polyomaviruses—exhibited low Ct values. qPCR and mNGS are fundamentally different methodologies and must be interpreted independently. qPCR results depend on the limit of detection and the viral copy numbers present in the samples. Samples with a higher viral load are more likely to be consistently detected by qPCR and should also be readily identified by mNGS. However, in our study, samples with low read counts showed low qPCR Ct values, particularly MWPyV. This discrepancy is likely to have been due to lower coverage, limited sequencing depth, and the presence of overrepresented sequences that may have been preferentially sequenced. Consequently, establishing cutoffs for acceptable numbers of sequence reads in Kraken-2-generated abundance data remains challenging. In such cases, careful interpretation of Kraken-2 abundance data is essential, alongside an advanced understanding of infections relevant to pediatric populations. For that reason, collaboration between researchers and clinicians is fundamental during interpretation of the obtained metagenomics data.

Across all patients, we observed a high overall abundance of commensal viruses belonging to the family *Anelloviridae*, which are not considered pathogenic [60]. In recent years, TTVs have been regarded as markers of immune system activation, as changes in viral load and replication capacity provide insights into the degree of host immunosuppression [61,62]. We hypothesize that the high density of *Anelloviridae* in all tested patients may be associated with altered immune function, which predisposes individuals to frequent viral activation and a high diversity of viral types. Given that the Shannon index did not indicate differences in *Anelloviridae* abundance between groups, we believe their presence is likely to have been incidental, as they are commonly detected in metagenomics analysis. Further studies focusing on anellovirus viral load in patients with FN could provide additional insights into their potential role in immune dysregulation.

Our study has several limitations related to the sampling process, the relationship between the identified viruses and clinical diagnoses, and its specific focus on viral causes. First, our study was based on a convenience sample with a small sample size. Consequently, this limits the generalizability of the findings and may have reduced the statistical power to analyze associations, potentially hindering the identification of risk factors. However, this study focuses on FN in children undergoing cancer treatment, a condition with relatively high prevalence in this population [63,64,65]. Therefore, the sample size was sufficient to detect differences between groups, and the results align with the existing literature. Moreover, our findings specifically focus on viral causes, and potential bacterial and fungal etiologies were not examined. Additionally, virus identification was performed using molecular methods such as metagenomics analysis and PCR, which do not necessarily correlate with the observed clinical symptoms, particularly FN. This was particularly evident, for example, in patient FN5, who tested positive by qPCR for EBV in plasma and saliva, CMV in plasma, and MWPyV in saliva. It remains unclear which of these viruses may have played a role in the febrile neutropenia or whether a causal relationship can be established.

The quantity of detected genetic material—whether indicative of a low or high viral load—as well as the number of viral reads could not be correlated with clinical symptoms or disease severity, due to the lack of established parameters. Furthermore, latent viruses, including the detected herpesviruses, can reactivate intermittently and persist for extended periods, especially in immunosuppressed patients. The detection of such sequences does not necessarily indicate disease progression, a causal role, or an impact on disease severity. Additionally, PCR and metagenomics analysis cannot distinguish between infectious virus particles and non-infectious viral genetic material processed by the immune system. Given these complexities, as well as the nature of FN, linking clinical presentation to a particular virus requires a multifaceted approach that integrates viral detection markers with patient-specific factors. Nevertheless, our study provides a broader perspective on the potential viral infectious causes of FN in children, extending beyond the traditionally considered bacterial factors.

## 5. Conclusions

In conclusion, this study aimed to characterize the blood and respiratory virome of pediatric oncology patients with FN and negative bacterial cultures. We identified a series of clinically important viruses, mainly belonging to the *Herpesviridade* family, which were directly validated by qPCR. An abundance of commensal viruses was also observed. Using mNGS, we identified viruses that have largely been neglected in clinical practice. Not only might these results improve the etiological diagnosis of FN, but they could also guide specific antiviral treatment where possible. Thus, identifying specific viral causes may reduce the recommended use of broad-spectrum antibiotics in FN and mitigate issues related to antibacterial resistance, which is particularly problematic in intensive care units where these patients are typically hospitalized and treated. Unfortunately, the etiological cause of most FN cases remains unknown. The detection of potential viral causes enhances FN diagnosis, facilitates the tailored administration of antiviral therapies, and potentially improves patient care. Furthermore, it underscores the need for further investigation into emerging or previously unsuspected viruses that may contribute to this severe condition.

## Figures and Tables

**Table 1:** General information regarding volunteer patients.

Group	Sample ID	Age (Years)	Clinical Diagnosis	Sex
Patients with febrile neutropenia	FN1	3	ALL * B, CNS ** relapse	M ******
	FN2	0.9	ALL B, CNS infiltration, IKZ deletion	M
	FN3	3	ALL B	M
	FN4	7	Hodgkin’s lymphoma	M
	FN5	2	ALL B	M
	FN6	4	ALL B	M
	FN7	8	ALL	M
	FN8	9	Anaplastic large cell NHL ***	M
	FN9	10	Adrenal carcinoma	F *******
	FN10	11	AML ****	F
	FN11	4	Langerhans cell histiocytosis	M
	FN12	4	Wilms tumor	F
	FN13	1	Infantile leukemia	M
	FN14	13	Congenital Dyskeratosis	M
	FN16	4	ALL B	M
Control group patients	FNC1	4	Metastatic alveolar rhabdomyosarcoma	M
	FNC4	10	Wilms tumor relapse	F
	FNC5	15	ITP *****	F
	FNC7	10	ALL B	F
	FNC8	4	Ganglioneuroblastoma	F
	FNC9	17	Germ cell tumor	F
	FNC10	7	T-cell lymphoblastic lymphoma	M
	FNC11	4	Burkitt lymphoma	M
	FNC12	15	Osteosarcoma	M
	FNC13	3	Wilms tumor	M
	FNC14	3	Retinoblastoma	M
	FNC15	12	Ewing’s sarcoma	M
	FNC17	7	Hodgkin’s lymphoma	M
	FNC19	8	ALL	M
	FNC21	2	Right eye retinoblastoma, E group	M

* ALL: Acute lymphoid leukemia; ** CNS: Central nervous system; *** NHL: Non-Hodgkin’s lymphoma; **** AML: Acute myeloid leukemia; ***** ITP: Idiopathic thrombocytopenic purpura; ****** M: Male; ******* F: Female.

**Table 2:** Primers and probes utilized for viral detection.

Virus	Primers and Probes	Sequence (5′-3′)	Concentration	Reference
Human Adenovirus	Forward primer (nested PCR, reaction 01)	GCCSCARTGGKCWTACATGCACATC	500 nM	[28]
	Reverse primer (nested PCR, reaction 01)	GCCSCARTGGKCWTACATGCACATC	500 nM	
	Forward primer (nested PCR, reaction 02)	GCCCGYGCMACIGAIACSTACTTC	500 nM	
	Reverse primer (nested PCR, reaction 02)	CCYACRGCCAGIGTRWAICGMRCYTTGTA	500 nM	
Herpes Simplex Virus 1	Forward primer	CATCACCGACCCGGAGAGGGAC	500 nM	[29]
	Reverse primer	GGGCCAGGCGCTTGTTGGTGTA	500 nM	
	Probe	FAM-CCGCCGAACTGAGCAGACACCCGCGC-TAMRA	185 nM	
Human Cytomegalovirus	Forward primer	ACCGTCTGCGCGAATGTTA	400 nM	[30]
	Reverse primer	TCGCAGATGAGCAGCTTCTG	400 nM	
	Probe	FAM-CACCCTGCTTTCCGAC-MGB	200 nM	
Human Herpes Virus 6	Forward primer	GAAGCAGCAATCGCAACACA	400 nM	[31]
	Reverse primer	ACAACATGTAACTCGGTG-TACGGT	400 nM	
	Probe	FAM-AACCCGTGCGCCGCTCCC-TAMRA	200 nM	
Human Herpes Virus 7	Forward primer	CGGAAGTCACTGGAGTAATGACAA	200 nM	[32]
	Reverse primer	ATGCTTTAAACATCCTTTCTTTCGG	200 nM	
	Probe	FAM-CTCGCAGATTGCTTGTTGGCCATG-TAMRA	100 nM	
Malawi Polyomavirus	Forward primer	TGAGAAGGCCCCGGTTCT	400 nM	[33]
	Reverse primer	GAGGATGGGATGAAGATTTAAGTTG	400 nM	
	Probe	FAM-CCTCATCACTGGGAGC-TAMRA	200 nM	
Washington University Polyomavirus	Forward primer	CCTGTTAGTGATTTTCACCCATGTA	400 nM	[34]
	Reverse primer	TGTCAGCAAATTCAGTAAGGCCTATATAT	400 nM	
	Probe	FAM-AAAGTTGTGTATTGGAAAGAACTGTTAGACA-TAMRA	100 nM	
Epstein–Barr virus	Forward primer	TCAACCTCTTCCATGTCACTGAGA	400 nM	[35]
	Reverse primer	TGGGTGAGCGGAGGTTAGTAA	400 nM	
	Probe	TCAGCCCCTCCACCAGTGACAATTC	200 nM	

**Table 3:** Quantitative sequencing data.

		Total Sequences	Sequences After Trimming	Classified Sequences	Viral Sequences	Non-Classified Sequences
Patients with febrile neutropenia	Plasma	522,460,676	410,262,663	182,791,640	11,622,377 (6.36%)	13,662,778
	Swab	467,728,618	312,924,626	52,164,628	1,659,562 (0.53%)	34,145,426
Control patients	Plasma	481,186,762	351,167,898	11,270,347	10,344,987 (2.95%)	2,084,891
	Swab	524,541,576	346,599,742	54,706,463	12,425,412 (3.58%)	49,774,976
Total		1,995,917,632	1,420,954,929	300,933,078	36,052,338 (11.98%)	99,668,071

**Table 4:** Characteristics of the viral agents detected among pediatric patients with febrile neutropenia.

ID	Sample Type	Detected Virus *	Cycle Threshold	Raw Read Number
FN1	Swab	Human herpesvirus 7	35.9	28
FN2	Swab	Epstein–Barr virus	33.4	27
FN5	Plasma	Human cytomegalovirus	36.99	7
	Swab	Epstein–Barr virus	31.81	2356
	Plasma	Epstein–Barr virus	37.15	2
	Swab	Malawi polyomavirus	25.98	3
FN7	Plasma	Cytomegalovirus	34.64	296
FN10	Swab	Human herpesvirus 6	36.95	7
	Swab	Human herpesvirus 7	36.96	72
FN12	Swab	Human herpesvirus 7	36.02	6
FN13	Swab	Human herpesvirus 6	34.38	2
FN14	Swab	Epstein–Barr virus	24.75	1,161,775
FN16	Swab	Human herpesvirus 6	35.57	83
	Swab	Human herpesvirus 7	34.68	1486
	Swab	Washington (WU) polyomavirus	34	17
FNC10	Swab	Human herpesvirus 6	35.81	2
	Swab	Human herpesvirus 7	33.61	9453

Legend: * Only viruses with clinical interest and detected by qPCR.

## Data Availability

The raw sequencing data obtained by next-generation sequencing have been deposited in the SRA database (NCBI) under the following accession number: PRJNA1219095.

## Abbreviations

The following abbreviations are used in this manuscript:

FN	Febrile neutropenia
ANC	Absolute neutrophil count
AML	Acute myeloid leukemia
ALL	Acute lymphoblastic leukemia
EBV	Epstein–Barr virus
HSV-1	Herpes simplex virus 1
hADV	Human adenovirus
SARS-CoV-2	Severe acute respiratory syndrome coronavirus 2
RSV	Respiratory syncytial virus
mNGS	Metagenomic next generation sequencing
HC-FMRP	Hospital das Clínicas, Faculty of Medicine of Ribeirão Preto
PCA	Principal component analysis
CMV	Cytomegalovirus
HHV	Human herpes virus
MWPyV	Malawi polyomavirus
WUPyV	Washington University polyomavirus
Ct	Cycle threshold
TTV	Torque teno virus
TTMV	Torque teno mini virus
TTMDV	Torque teno midi virus
